# Differing susceptibility of C57BL/6J and DBA/2J mice—parents of the murine BXD family, to severe acute respiratory syndrome coronavirus infection

**DOI:** 10.1186/s13578-021-00656-8

**Published:** 2021-07-19

**Authors:** Kui Li, Yang Shen, Mark A. Miller, Jennifer Stabenow, Robert W. Williams, Lu Lu

**Affiliations:** 1 Department of Microbiology, Immunology and Biochemistry, University of Tennessee Health Science Center, Memphis, TN 38163 USA; 2 Regional Biocontainment Laboratory, University of Tennessee Health Science Center, Memphis, TN 38163 USA; 3 Department of Genetics, Genomics and Informatics, University of Tennessee Health Science Center, Memphis, TN 38163 USA

**Keywords:** Severe acute respiratory syndrome coronavirus, Coronavirus disease-2019, SARS-CoV-2, C57BL/6J, DBA/2J, BXD family, Systems genetics, Viral replication, Weight loss, Susceptibility

## Abstract

The ongoing coronavirus disease-2019 (COVID-19) pandemic, caused by a novel coronavirus termed severe acute respiratory syndrome coronavirus-2 (SARS-CoV-2) that is closely related to SARS-CoV, poses a grave threat to global health and has devastated societies worldwide. One puzzling aspect of COVID-19 is the impressive variation in disease manifestations among infected individuals, from a majority who are asymptomatic or exhibit mild symptoms to a smaller, largely age-dependent fraction who develop life-threatening conditions. Some of these differences are likely the consequence of host genetic factors. Systems genetics using diverse and replicable cohorts of isogenic mice represents a powerful way to dissect those host genetic differences that modulate microbial infections. Here we report that the two founders of the large BXD family of mice—C57BL/6J and DBA/2J, differ substantially in their susceptibility to a mouse-adapted SARS-CoV, MA15. Following intranasal viral challenge, DBA/2J develops a more severe disease than C57BL/6J as evidenced by more pronounced and sustained weight loss. Disease was accompanied by high levels of pulmonary viral replication in both strains early after infection but substantially delayed viral clearance in DBA/2J. Our data reveal that the parents of the BXD family are segregated by clear phenotypic differences during MA15 infection and support the feasibility of using this family to systemically dissect the complex virus-host interactions that modulate disease progression and outcome of infection with SARS-CoV, and provisionally also with SARS-CoV-2.

Dear Editor,

The sudden emergence of a novel coronavirus (CoV), termed severe acute respiratory syndrome coronavirus-2 (SARS-CoV-2) [1] in late 2019, is a sobering reminder that CoVs can cause life-threatening respiratory illnesses, as were also witnessed by the 2002–2003 SARS-CoV epidemic and the Middle East Respiratory Syndrome (MERS)-CoV outbreaks that first surfaced in 2012. These three highly pathogenic human CoVs (hCoVs) have upended the traditional view of hCoV pathogenesis established with four other hCoVs (hCoV-229E, hCoV-OC43, hCoV-NL63 and hCoV-HKU1), which are typically associated with common cold or mild respiratory tract infections. Although hCoVs have been discovered for almost six decades, much remains to be learned regarding the molecular details by which this family of unusually large, enveloped RNA viruses interact with their human host. Of the many unanswered questions, two stand out most frequently: (1) what account for the high virulence of SARS-CoV, SARS-CoV-2 and MERS-CoV, in contrast to their common cold-causing relatives, in humans, and (2) why only a minority of individuals infected with SARS-CoV-2 suffer serious to even deadly progression whereas most others exhibit mild or in some cases, no symptoms. A better understanding of the factors that regulate pathogenesis and outcome of hCoV infections is critical for informing the development of countermeasures and for identifying high-risk patient populations in need of heightened medical attention, especially when medical resources are overwhelmed during a pandemic like COVID-19 that is still raging.

First emerged in December 2019, in Wuhan, China, SARS-CoV-2 was associated with mysterious, severe pneumonia cases in that city [2]. The virus spread rapidly across the globe in just a few months, causing a pandemic disease known as coronavirus disease-2019 (COVID-19). As of April 29, 2021, more than 149 million people worldwide have been infected and the death toll has surpassed 3 million (WHO). Unfortunately, no effective antiviral treatments are available, leaving supportive care as the main, if not sole, option for managing hospitalized COVID-19 patients. Several vaccines under emergency use authorization have been rapidly deployed and helped slow the transmission of SARS-CoV-2 in a handful of countries but are not readily accessible to many other parts of the world. Adding to these worries is the recent emergence of new viral variants of concern, which could outpace and defeat the massive vaccination campaigns that are underway. Identifying infected individuals predisposing to more severe disease is critical for saving lives by prioritizing precious medical resources that are overtaxed in the middle of an epidemic of unprecedented magnitude. Yet, little is known as to why ~ 80% of SARS-CoV-2-infected individuals exhibit little or only mild symptoms while merely a small fraction (0.001–8.3%) succumbs to the infection that is largely age-dependent [3]. As in the case of SARS, COVID-19 patients who are elderly or with pre-existing medical conditions are at increased risk for severe outcomes [2]. However, exceptions are not uncommon, suggesting other factors are also at play. Conceivably, variations in host genetic makeup most likely regulate the differential susceptibility to and outcome of SARS-CoV-2 infection.

SARS-CoV-2 shares ~ 79% sequence homology with the original SARS-CoV. Both viruses utilize the angiotensin-converting enzyme 2 (ACE2) as a receptor for entering susceptible host cells and initiating viral replication cycle [2]. Understanding pathogenesis of SARS-CoV and SARS-CoV-2 depends on small, tractable animal models. While laboratory mice in general are not susceptible to the original isolates of either virus because of the weak affinity of mouse ACE2 to viral Spike glycoprotein, a mouse-adapted SARS-CoV, dubbed MA15, was developed by serially passaging the human Urbani strain of SARS-CoV in the lungs of BALB/c mice. When given intranasally, MA15 inflicts severe pulmonary infection in young adult BALB/c mice, resembling many pathological features of human SARS [4]. By using MA15 infection in various laboratory mouse strains, it has been demonstrated that T cell responses mediate protection from clinical illness and promotes viral clearance, while deletion of several key innate immune signaling molecules, such as MYD88 or STAT1 [5], leads to more severe disease. However, these likely represent the tip of the iceberg when it comes to the full picture of host gene pathways/networks that dictate susceptibility to and disease severity of SARS-CoV infection, much of which remains to be elucidated. Although mouse-adapted SARS-CoV-2 viruses have recently been described, they are yet to be made widely available in the field.

Human diseases are known to result from complex interactions between genetic and environmental factors. Among the suitable mammalian models for studying genetic heterogeneity and complex gene-by-environment interactions in pathogenesis, the recombinant inbred (RI) BXD strain family represents the best characterized mouse genetic reference panel and has been widely used as a platform to study the genetic basis for various disorders, including the genetics of immune function and infectious diseases. Descended from crosses between C57BL/6J (referred to as B6) and DBA/2J (referred to as D2) inbred strains, the RI BXD strain set comprises ~ 150 fully sequenced inbred strains and segregates for ~ 6 million sequence variants scattered across the genome, close to the number of single nucleotide polymorphisms segregating in human populations. As such, this large genotype set affords high statistical power and high mapping precision (a maximal resolution of < 0.5 Mb across the genome), making them especially suitable to define gene variants linked to complex genetic diseases [6]. Of note, the BXD parents B6 and D2 are known to differ in numerous genetic, metabolic, and immunologic aspects, all of which interact and may contribute, albeit to varying extent, to distinct disease phenotypes. Specifically relevant to infectious diseases and immune-mediated ailments, it is worth highlighting a few examples of the variations in immune genes/responses and distinctions in sensitivity to pathogens between B6 and D2 mice. Whereas D2 carry a loss-of-function mutation in complement component 5 (C5) due to a frame shift in their *﻿Hc* gene, B6 have intact C5. Compared with B6 having a Th1-biased adaptive immune response to microbial pathogens, D2 are more biased toward a Th2-predominant response. The BXD founders have also been shown to differ starkly in susceptibility to various influenza virus strains, with B6 resistant to virally induced morbidity and mortality while D2 highly susceptible. Strikingly, the lethal dose of a highly pathogenic H5N1 influenza virus for B6 was found to be ~ 4 logs higher than that for D2. Based on and motivated by these prior observations, the BXD strains have been successfully employed to map genetic elements (quantitative trait loci, QTL) that control influenza virus resistance. However, whether this family can be leveraged to interrogate CoV-host interactions critical for pathogenesis of SARS or COVID-19 has not been reported. Intriguingly, the Baric group recently used the Collaborative Cross set to identify several QTLs that may underpin different phenotypes in SARS-CoV infection, lending support for the value of mouse genetic reference families in dissecting aspects of CoV pathogenesis [7].

To determine whether the BXD family is suitable as a diversity model to define causal molecular networks modulating SARS pathogenesis and disease severity, we investigated whether the two founders, B6 and D2, differ significantly in susceptibility to MA15 infection. We first examined our existing transcriptomic data on the B6 and D2 parents and found that basal pulmonary levels of viral entry receptor *Ace2* transcript are well matched [8], ruling out the possibility of pre-infection *Ace2* expression as a confounding factor. We then challenged groups of 10-week old B6 (*n* = 26) and D2 (*n* = 28) via the intranasal route with 10^5^ TCID_50_ of MA15 virus (diluted in 50-μl phosphate-buffered saline, PBS), and monitored weight changes for 9 days. On each day for the first five days post infection (dpi), we sacrificed a subset of infected mice (3–5/group) and harvested their lungs for evaluating viral loads in lung homogenates and did so with the remaining mice on day 9 (end point). As controls, we also inoculated 4 mice per strain with culture supernatant of Vero-E6 cells (the cell host for propagating the MA15 virus stocks) diluted in PBS. As shown in Fig. 1 (dashed lines), neither B6 nor D2 receiving control inoculum (i.e., mock infection) lost weight during the 9-day period, indicating that the light anesthesia (isoflurane at a concentration of 3–5% for induction, then reduced to 2% for maintenance during intranasal infection) and intranasal challenge procedures do not produce appreciable adverse effects, nor do ingredients of the culture medium for Vero-E6 cells. In contrast, there was significant weight loss in both strains following MA15 infection. On average, B6 lost a little over 10% of body weight by 2 to 3 dpi, followed by a rapid recovery beginning on day 4. On 8–9 dpi, all B6 mice had regained weight to nearly pre-infection levels (Fig. 1, solid black line). This weight change pattern observed with MA15-infected B6 mice is similar to that reported by Sheahan et al. [9]. In comparison, D2 had substantially greater weight loss from 1 to 5 dpi than B6 (*p* < 0.05 for days 1–4, Students *t*-test), and these mice did not start to recover until 6 dpi. Maximal weight loss in infected D2 occurred on 3–4 dpi, and approached an average of 18% (Fig. 1, solid purple line). Notably, two D2 cases died on 3 and 5 dpi, after losing ~ 18% and ~ 25% weight, respectively. Analyses of infectious viral titers in lung homogenates, by fifty-percent tissue culture infective dose (TCID50) assay on permissive Vero-E6 cells [10], from a subset of infected mice revealed high levels of pulmonary viral replication in both strains during the first 4 days post infection (Fig. 2), with D2 strain harboring consistently 2–3-fold higher levels of viral load, although the differences did not reach statistical significance. Interestingly, while lung viral titers had dropped to the 10^3^ TCID_50_/g tissue range in B6 on 5 dpi (a ~ 15,000-fold decrease from day 1), viral load remained relatively high (at > 10^5^ TCID_50_/g levels) in D2 (a ~ 400-fold decrease from day 1). The ~ 62-fold higher lung pathogen load in D2 than in B6 (p < 0.05, Students t-test) at this time point was consistent with a sustained weight loss in D2 on 5 dpi. (Fig. 1, solid purple line). Of note, when sacrificing infected mice for tissue collection, we consistently observed higher frequency of lung lobe consolidation in D2 than in B6. Additional experiments specifically examining gross dissection and histopathology of lungs of MA15-infected mice from both strains in statistically significant numbers will be needed to confirm this intriguing observation. Nevertheless, no virus could be detected in lungs on 9 dpi in either strain, a time at which all remaining mice in the infected groups had regained weight to almost pre-infection levels.

**Fig. 1:**
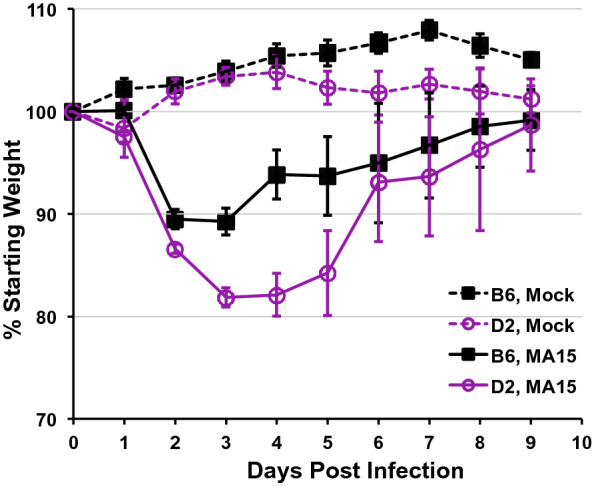
Weight change of B6 and D2 mice following infection by a mouse-adapted SARS-CoV, MA15. B6 (n = 26) and D2 (n = 28) mice were inoculated with 10^5^ TCID_50_ of MA15 virus in PBS via the intranasal route. Control mice (Mock, 4/group) received Vero-E6 culture supernatant diluted in PBS. Mice were monitored daily for weight change (expressed as % starting weight, Mean ± SEM) and a subset of infected mice (3–5/group) were sacrificed on 1, 2, 3, 4, 5, and 9 dpi for measuring viral titers in the lungs (see Fig. 2)

**Fig. 2:**
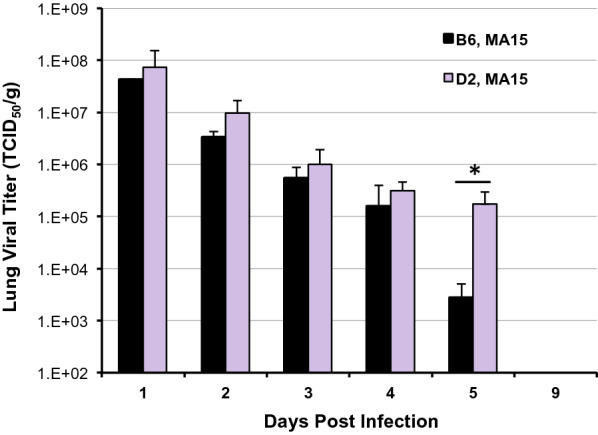
Viral titers in the lungs of B6 and D2 mice intranasally challenged with 10^5^ TCID_50_ of MA15 virus. Data shown are infectious viral titers per gram lung tissue (expressed as mean viral titer ± SD) from 3–5 mice/strain at each time point. Asterisk denotes p < 0.05 between B6 and D2 on 5 dpi

MA15 is highly lethal in the BALB/c strain [4]. To our surprise, we observed few deaths in MA15-infected D2 mice and the virus was even less virulent in B6. We wondered whether our infection dose of 10^5^ TCID_50_ was not high enough. To address this, we infected new cohorts of B6 (*n* = 11) and D2 (*n* = 10) with an increased dose of 5 × 10^5^ TCID_50_. For this experiment we continually monitored all cases for weight loss without sacrificing animals for tissue collection. At this high infection dose, B6 lost an additional 5% (on average) of body weight compared to the lower dose challenge (15% vs 10%) on 2 dpi but the overall weight change kinetics followed a similar track with weight dipping to their lowest points on 2–3 dpi, followed by a rapid rebound starting on 4 dpi. Likewise, by 8–9 dpi average weight of infected B6 had recovered to pre-infection levels (Fig. 3, solid black line). Another notable difference was that 2 of the 11 infected B6 cases ended with a lethal outcome (both deaths occurred on 5 dpi; one died and the other was scored as "dead" due to losing > 25% weight, our criterion for euthanasia). In the D2 infection group, maximal average weight loss remained at ~ 18% and occurred on 3–4 dpi. (Fig. 3, solid purple line), similar to those observed in the lower dose challenge experiment. However, weight loss was substantially prolonged and average weight of infected D2 cases hovered around 84–86% on 5–7 dpi before slowly rebounding on 8 dpi. Intriguingly, D2 infected at this high dose did not acquire their weight back to > 95% starting weight until after 18 dpi. Students *t*-test analyses suggest that the extent of weight loss in infected B6 and D2 cases differed significantly for most of the time points (*p* < 0.05, on 1, 3–16, and 20 dpi). Of note, in this experiment 3 of the 10 infected D2 cases succumbed between 3 and 7 dpi. Thus, although MA15 appears to be slightly more lethal to D2 than to B6 (3/10 vs. 2/11), the majority of animals of either genetic background present a non-lethal disease, even when infected at a high dose.

**Fig. 3:**
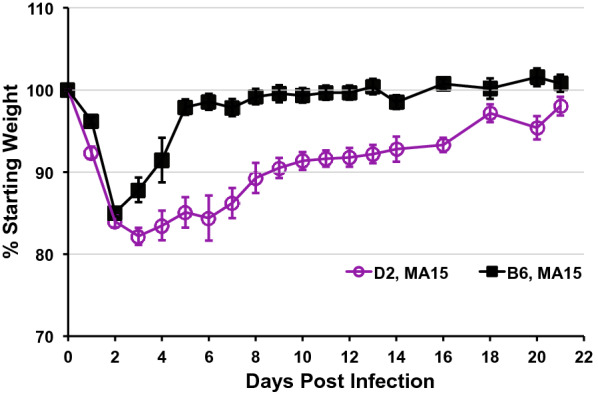
Weight change of B6 (n = 11) and D2 (n = 10) mice following intranasal challenge with high dose (5 × 10^5^ TCID_50_) of MA15 virus. Data are expressed as % starting weight, Mean ± SEM for each time point

Collectively, data from these experiments demonstrate that D2 strain is substantially more susceptible to infection by MA15 virus than B6 mice, with the former developing a more severe disease (more pronounced and sustained weight loss) and supporting more prolonged viral replication in the lungs than the latter. It is thus reasonable to hypothesize that the RI BXD family of mouse strains represent a promising platform for forward systems genetics probing SARS-CoV interactions with the host. Unlike conventional scientific investigations that typically begin with a specific hypothesis, systems genetics using diverse and replicable cohorts of BXD mice is an unbiased approach that enables the delineation of a fuller picture of candidate host genes, pathways or polymorphisms that influence susceptibility/resistance to and/or pathogenesis of SARS-CoV infection. Because a majority of infected mice could survive the infection, be they of D2 or B6 background, death/survival cannot be used as a reliable phenotypic readout to distinguish between the two mouse strains, at least when MA15 is used as the challenging pathogen. On the other hand, these studies have identified pathogen load (i.e., viral titers in the lungs) on ~ 5 dpi and % weight loss on ~ 3–5 dpi as distinct phenotypes for QTL mapping of host resistance genes against SARS-CoV in the BXD genotype panel. No less important, more in-depth characterization of the differential host responses of B6 and D2 to SARS-CoV, e.g., by OMICs approaches, may lead to additional markers/phenotypes that are worthy of investigation. The next step would be to interrogate the family of RI BXD strains for their varying responses to MA15 virus challenge, using the various measurable phenotypic differences identified between the infected parental strains as readouts. Together with large-scale transcriptomics and proteomics profiling of lungs from sham- and MA15-infected RI BXDs, these future experiments will glean comprehensive phenotypic and OMICs data sets across genetically diverse BXDs, which can then be used for QTL mapping and computational modeling of genetic factors that modulate SARS-CoV infection and pathogenesis.

Precisely what factor(s) may account for the differing susceptibility of the parental B6 and D2 strains to MA15 infection will remain elusive until the BXD studies, as outlined above, have been conducted. In mouse models of SARS-CoV infection, the pathogen is cleared within days in animals surviving the viral challenge. The innate antiviral immune responses, characterized by the induction and signaling of interferon (IFN) family of antiviral cytokines, have been suggested to play an important role in resolving MA15 infection in mice. We surveyed our lung and spleen Illumina transcriptomics data from uninfected B6 and D2 mice for differences in expression of a subset of host genes in the IFN antiviral signaling pathway, to seek possible clues. Included on this list were three viral RNA sensors Ifih1, Ddx58, and Tlr3 and their adaptors Mavs (a.k.a., D430028G21Rik) and Ticam1, two key transcription factors Irf3 and Irf7, as well as several genes critical for IFN autocrine/paracrine signaling including Ifnar1, Ifnlr1 (a.k.a., Il28ra), Stat1, and Stat2. Even when using a low-threshold cutoff value, i.e., 1.5-fold, we did not find expression of any of these genes was substantially different between B6 and D2, either in the lungs or spleen (Table 1), suggesting these well-studied genes in innate immune sensing of and IFN antiviral responses to viruses are unlikely responsible for the observed differential susceptibility to MA15 infection. However, we caution that it remains unclear in the context of SARS-CoV infection, whether activation and/or propagation of IFN antiviral responses are differentially regulated between B6 and D2 mice, and whether the more severe disease in D2 than in B6 results from an exacerbated, harmful inflammatory reaction. Additionally, there is the possibility that, genes and pathways not directly involved in antiviral or inflammatory responses contribute significantly. Systems genetics of MA15 infection in the RI BXD family of mice will provide an unbiased way to examine all these possibilities and lend important insights.

**Table 1:** Comparison of the expression of select innate immune genes in the IFN signaling pathway in the lungs and spleen between B6 and D2 mice

Gene ID	Gene symbol	Description	Log2 diff. in lungs (B6-D2)	Log2 diff. in spleen (B6-D2)
230073	Ddx58	DExD/H-box helicase 58	− 0.066	0.438
71586	Ifih1	Interferon induced with helicase C domain 1	− 0.058	0.411
142980	Tlr3	Toll-like receptor 3	− 0.390	0.358
228607	D430028G21Rik	Mitochondrial antiviral signaling protein	0.161	− 0.094
106759	Ticam1	Toll-like receptor adaptor molecule 1	0.153	0.008
54131	Irf3	Interferon regulatory factor 3	− 0.062	0.089
54123	Irf7	Interferon regulatory factor 7	− 0.205	0.007
15975	Ifnar1	Interferon (alpha and beta) receptor 1	− 0.088	0.044
242700	Il28ra	Interleukin-28 receptor subunit alpha	− 0.162	− 0.021
20846	Stat1	Signal transducer and activator of transcription 1	− 0.457	0.256
20847	Stat2	Signal transducer and activator of transcription 2	− 0.212	− 0.553

None of the genes listed herein showed a difference in mRNA expression of ≥ 1.5-fold (i.e., 0.58 in Log2) between B6 and D2 mice in the lungs or spleen. D430028G21Rik is also known as Mavs; Il28ra is also known as Ifnlr1; Ticam1 is also known as Trif

Last but not the least, given the many similarities between SARS-CoV and SARS-CoV-2, studies are warranted to determine how much the distinct phenotypes in host responses to SARS-CoV MA15 infection between B6 and D2 reported herein can be replicated using mouse-adapted SARS-CoV-2 strains that may soon become widely available, and the extent to which the now greatly expanded BXD family [6] can be leveraged to interrogate crucial host-virus interactions that may be targeted for therapeutic interventions against COVID-19.

## Data Availability

All data generated or analyzed during this study are included in this article.

## Abbreviations

COVID-19: Coronavirus disease-2019
SARS: Severe acute respiratory syndrome
MERS: Middle East respiratory syndrome
hCoV: Human coronavirus
ACE2: Angiotensin-converting enzyme 2
RI: Recombinant inbred
QTL: Quantitative trait loci
PBS: Phosphate-buffered saline
dpi: Days post infection
TCID_50_: Fifty-percent tissue culture infective dose
